# Spectral–Entropy Network Analysis of Multidimensional Poverty: An Explainable AI Framework for Complex Socioeconomic Systems

**DOI:** 10.3390/e28070746

**Published:** 2026-07-01

**Authors:** Sadullah Çelik, Cemile Zehra Köroğlu, Muhammet Ali Köroğlu

**Affiliations:** 1 Department of International Trade and Finance, Aydın Adnan Menderes University, Aydın 09000, Türkiye; sadullah.celik@adu.edu.tr; 2 Department of Social Work, Uşak University, Uşak 64000, Türkiye; muhammet.koroglu@usak.edu.tr

**Keywords:** multidimensional poverty, explainable artificial intelligence, SHAP, spectral–entropy network analysis, complex socioeconomic systems, I32, O15, C45, C63, D63

## Abstract

Multidimensional poverty is a socioeconomic problem that results from nonlinear interdependencies of various socioeconomic indicators such as educational, health, and living standards indices. This paper considers an XAI-based approach for studying the structural topology and dependency structures of multidimensional poverty systems. It relies on a combination of machine learning approaches, network science, spectral graph theory, and entropy-based complexity measures for revealing the systemic interdependencies between different poverty indicators. The structure of interdependencies between socioeconomic indices associated with education is represented by the multilayer perceptron (MLP). The interpretability of the model is provided via the computation of SHAP values by means of the KernelSHAP method. Higher-order interactions between variables are revealed via the construction and analysis of SHAP-based interaction networks. The proposed methodology is then employed on the GEMPI 2025 dataset consisting of 109 countries. The results show significant consistency in the structural map (R^2^ = 0.9890) in combination with stable internal consistency in cross-validation (R^2^ = 0.9864, SD = 0.0074). The SHAP analysis shows that standards of living and health have a high influence on the structural mapping of education, while the contribution of income-related features is lower compared to other features. Entropy analysis points toward partially fragmented dependency networks with a moderate concentration of explanatory influences (H = 1.705). The proposed framework can be used to characterize structural dependencies, identify influencing system components, and map informational processes in multidimensional poverty systems by combining the methodology of explainable artificial intelligence with entropy–spectral network analysis.

## 1. Introduction

The concept of multidimensional poverty has emerged as one of the important issues regarding development in economics and mathematics. It is imperative to formulate tools for analyzing complex and interdependent structures effectively. As opposed to other methodologies that use monetary measures, MPI measures deprivations in multiple dimensions—namely, health, education, and standards of living—and it provides a structured analysis of multidimensional poverty [1,2,3,4]. Although popular, the Alkire–Foster methodology, based on the idea of multidimensional poverty, does not adequately capture complex nonlinear interdependencies among deprivations because it focuses on aggregating dimensions based on incidence and intensity. On the contrary, the idea of interdependency between these deprivations fits perfectly with the concept of human capital theory [5], according to which education, health, and income interact and influence each other. Moreover, it corroborates the idea of credit constraints theory [6], which postulates the persistence of poverty due to credit constraints for financial and educational opportunities. In line with these theoretical frameworks, it seems unreasonable to treat deprivation variables separately rather than as a single system.

In recent years, empirical evidence suggests that the causes of poverty are not independent but rather interrelated and mutually reinforcing forms of deprivation, which vary from one country to another and regionally [7,8]. The existence of different types of deprivation implies that multidimensional poverty should be considered a high-dimensional system with hidden variables and interactions. At the same time, current methodologies for addressing this phenomenon are mostly based on descriptive statistics and regression, thus making them inherently restricted in their ability to capture nonlinearity and interaction effects. Modern research within development economics and welfare economics reinforces this view through the emphasis on feedback mechanisms between education, health, and income dynamics [9,10].

The problem can be approached using a network–theoretic approach in which the relations among variables of poverty can be naturally mapped as networks in which the variables themselves are considered as nodes, whereas their interdependencies may be considered as the edges. However, there is not much literature in the study of the topological and spectral properties of such systems within the context of poverty. Spectral graph theory is a special area of graph theory that enables one to analyze complex systems through the power of mathematics [11,12,13,14,15]. Within this paradigm, network-based economic modeling has attracted considerable interest as an approach to understanding systemic interdependencies within socioeconomic systems, but it has not yet received much application in the field of multidimensional poverty.

Concurrently, there have been several approaches in the field of machine learning that have proven to be very effective in handling nonlinear and highly dimensional socioeconomic data. Even though such machine learning algorithms have performed well in terms of structural explanation, their poor interpretability makes it hard to employ them in the context of policies. XAI and the implementation known as SHAP (SHapley Additive Explanations) are methods that help with overcoming this problem through cooperative game theory and explaining the predictions of any machine learning model [16]. Nonetheless, most studies have concentrated on prediction/fit performance or interpretability rather than both structural and topological representations of feature interactions [4,17,18,19,20].

Therefore, the first research gap this paper uncovers is the absence of a universal mathematical–economic system that simultaneously encompasses nonlinear dependencies, interpretation capabilities, and the topology of multidimensional poverty phenomena.

In order to address this gap directly, this research relies on a systems economics viewpoint, under which poverty is considered a consequence of interactions between human capital, institutions, and limited resources. Such an approach enables incorporating economic theory and computation together.

Contrary to those studies focusing only on one methodology, the novelty in this paper is methodological and theoretical rather than algorithmic. In fact, unlike other papers, this paper does not develop any new architecture or graph algorithm for machine learning. Instead, it provides an economic-theory-guided integrative approach uniting traditional economic mechanisms with modern computational techniques into a single analysis chain.

They comprise exploratory data analysis coupled with PCA for the identification of dimensional structure, nonlinear ML modeling (MLP) for modeling interaction effects, a SHAP-based approach for explaining results, and spectral graph theory for analyzing the topological and structural features of networks. The revolutionary point of using this combination lies in the possibility of translating model-level explanation (expressed through SHAP values) into the language of networks. In other words, there is a bridge between the output of statistical learning and the economic reality described by the former.

Using such an approach, one can explore various dimensions of multidimensional poverty at once, such as latent structures, nonlinearity, structural causality, and topological organization.

This problem is addressed by this research by proposing an innovative analytical framework that integrates PCA, nonlinear MLP modeling, SHAP explainability, and spectral graph theory. Within the proposed analytical framework, the dimension of education deprivation (EC) will be introduced as a component of the structural model of dimensions. Therefore, it will be possible to conduct a mathematical investigation of its interaction with other dimensions.

The originality and contributions of this paper are defined by its ability to connect two distinct approaches—predictive modeling and structural analysis—within the same mathematical approach. The first step involves quantifying the interactions between features through SHAP-based explainability. Then, the next step consists of rephrasing these interactions in terms of a weighted graph representation. Entropy methods are employed to quantify how distributed and concentrated the explanation power of SHAPs is for each feature. Finally, SHAP values at the observation level are used to measure the stability of feature importance across observations. Overall, the combination of these four steps defines a novel “explain–embed–analyze” framework that was never proposed before in any of the existing multidimensional poverty literature.

Under this approach, the purpose of this paper is to address the following important questions regarding the problem under investigation. (i) What is the composition of the key determinants of EC from a structural perspective? (ii) How do the identified structural elements influence each other through a nonlinear interdependent mechanism? (iii) What can spectral analysis, network approaches and entropy-based techniques reveal regarding the structure of poverty?

This paper contributes to the existing body of knowledge on multidimensional poverty analysis and complex socioeconomic systems by offering an economically motivated methodology of analyzing multidimensional poverty based on machine learning and explainable AI techniques along with network approaches. Thus, the innovation of this paper cannot be reduced to the independent creation of algorithms for solving particular tasks; rather, it lies the synthesis of network-based methods and computer algorithms in accordance with economic considerations of multidimensional poverty as a complex socioeconomic phenomenon.

## 2. Materials and Methods

**Figure 1:**
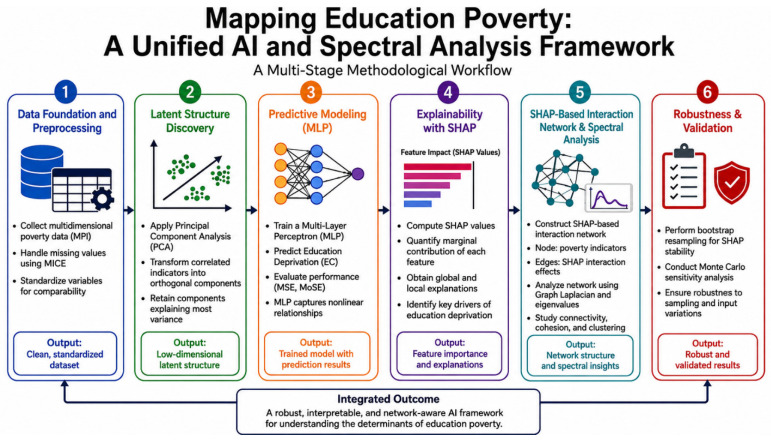
Mapping education poverty: a unified AI and spectral analysis framework.

In this paper, the proposed methodology comprises a multi-phase analysis approach which incorporates the preprocessing of the data, dimensionality reduction, nonlinear predictive models, explainable artificial intelligence, and network-based spectral analysis techniques for analyzing multidimensional poverty. This entire process is described in Figure 1.

The first phase consists of data collection from the global multidimensional poverty index database and then missing data imputation through MICE along with normalization. This process is shown in Figure 1.

In the second stage, the PCA approach is used to discover underlying structures and perform dimensional reduction by retaining most of the variance, leading to a low-dimensional representation of multidimensional poverty.

The third stage involves the use of an MLP model to predict education deprivation (EC). The effectiveness of the prediction will be measured in terms of error statistics and the comparison of its performance against other machine learning techniques.

The fourth stage will use the explainable AI technique based on SHAP values to understand predictions made by the model from global and local perspectives by calculating contributions of features.

Lastly, the fifth stage uses SHAP to develop interaction networks and uses spectral graph theory to explore the system structure. Figure 1 shows the relationship between prediction and structural analysis in this stage through the Laplacian eigenvalues.

Robustness will be established through bootstrap sampling and Monte Carlo sensitivity analysis.

Figure 1 below presents the integration of prediction, explainability, and structure within one framework for analyzing multidimensional poverty dynamics.

### 2.1. Data and Variables

For this paper, the secondary cross-sectional database used in the analysis was selected from the report prepared by the United Nations Development Programme called the Global Multidimensional Poverty Index (Global MPI) 2025 [21]. This database consists of estimates of the national multidimensional poverty of 109 countries and has been prepared based on standard data from household surveys. As the Global MPI database serves as an official international database to measure multidimensional poverty, its use for structural analysis has been deemed appropriate.

Let i=1,2,…,N be the country with t representing the year of the survey. The variable of interest is the Multidimensional Poverty Index, $MPI_{it}$. It can be defined as shown in Equation (1).

(1) $$MPI_{it}=H_{it}\times A_{it}$$

In Equation (1), $H_{it}$ denotes the headcount ratio, which is defined as the proportion of multidimensionally poor people. On the other hand, $A_{it}$ denotes the deprivation intensity, which is defined as the average proportion of deprivation experienced by poor people. The headcount ratio is given by Equation (2).

(2) $$H_{it}=\frac{q_{it}}{n_{it}}$$

In Equation (2), $q_{it}$ denotes the multidimensionally poor people’s count, whereas $n_{it}$ denotes the population count.

There is other information in the dataset that includes inequality among the poor $\left(I_{it}\right)$, severe multidimensional poverty $\left(SMP_{it}\right)$, and population that is vulnerable to multidimensional poverty $\left(VMP_{it}\right)$. Moreover, MPI has three dimensions: health $\left(HC_{it}\right)$, education $\left(EC_{it}\right)$, and living standards $\left(LSC_{it}\right)$.

In addition, the dataset has poverty-level indicators based $\left(LSC_{it}\right)$ on income levels, such as the national poverty line $\left(NPL_{it}\right)$ and the USD 3 a day PPP poverty rate $\left(PPP3_{it}\right)$.

In this case, the dependent variable of interest for the use of supervised machine learning algorithms is the Education Component $\left(EC_{it}\right)$ rather than the overall Multidimensional Poverty Index $MPI_{it}$. Thus, the purpose of the analysis is not to forecast MPI values but rather to analyze the structure of education deprivation within the multidimensional poverty framework.

In this regard, the application of machine learning should be interpreted as an exploratory investigation of the structure of dependency of the given poverty measures rather than as an out-of-sample prediction task. As follows from the mathematical character of dependency in Alkire–Foster measures, the results obtained from models will produce meaningful dependencies.

All variables that were employed in this research are presented in Table 1 below.

The dataset can be expressed mathematically and represented as a data matrix. For instance, let us assume that the dataset matrix is given by X, where $X\in R^{N\times K}$, and N=109 is the number of countries and K is the number of observed variables. The matrix form of the dataset is given by Equation (3). Even though there are only 109 countries in the dataset, it constitutes the full set of countries in the Global MPI 2025 dataset. Thus, the use of the MLP method to identify nonlinear associations between the factors and to build an explanation for SHAP was justified.

(3) $$X=\left[\begin{array}{ccccccccccc} MPI_{1} & H_{1} & A_{1} & I_{1} & SMP_{1} & VMP_{1} & HC_{1} & EC_{1} & LSC_{1} & NPL_{1} & PPP3_{1}\\ MPI_{2} & H_{2} & A_{2} & I_{2} & SMP_{2} & VMP_{2} & HC_{2} & EC_{2} & LSC_{2} & NPL_{2} & PPP3_{2}\\ \vdots & \vdots & \vdots & \vdots & \vdots & \vdots & \vdots & \vdots & \vdots & \vdots & \vdots \\ MPI_{N} & H_{N} & A_{N} & I_{N} & SMP_{N} & VMP_{N} & HC_{N} & EC_{N} & LSC_{N} & NPL_{N} & PPP3_{N} \end{array}\right]\;\;\;\;\;\;$$

For example, in Equation (3), the rows in the matrix stand for the countries, whereas the columns in the matrix stand for the multidimensional poverty indicators in the dataset. This type of data arrangement suits well the application of numerous statistical modeling techniques, machine learning models, and even XAI. Both the dataset and the Python 3.14 codes used in this paper are released to make the research findings transparent to the public. On this account, it is emphasized that all of the data processing techniques, modeling techniques, and analysis techniques used in this paper can be accessed from the following GitHub repository link: https://github.com/Sadullah4535/Multidimensional-Poverty-Indicators-/ (accessed on 25 March 2026). It is pointed out in this regard that the whole framework of this paper can be replicated by the interested researchers from the aforementioned GitHub repository link.

The set of features to be used in this paper was selected according to the guidelines given by the UNDP Global Multidimensional Poverty Index to ensure theoretical consistency and multidimensionality. Random feature deletion was not utilized; all the features were retained to include poverty aspects such as severity of deprivation, extreme poverty, vulnerability, health, education, standard of living, and income poverty. Whether a feature was necessary or important for the analysis was later ascertained through latent structure analysis (PCA—Section 3.2) and SHAP value analysis (Section 3.4 and Section 3.5).

### 2.2. Preprocessing and Mathematical Transformation

The dataset presented in Section 2.1 has N=109 countries, including various poverty-related indicators. However, some of the indicators have missing information, specifically for inequality among the poor $I_{it}$, national poverty line $NPL_{it}$, and PPP-based poverty rate $PPP3_{it}$. Therefore, the dataset can be represented as follows: $X\in R^{N\times K}$. The missing information can be defined as shown in Equation (4).

(4) $$X_{ij}\notin R,\;\;\mathrm{for}\;\mathrm{some}\;\left(i,j\right)$$

This implies that some of the elements in the data matrix are not observed.

Before the process of imputation, missing values were noted only for the NPL variable, which had 7 missing data points equaling about 0.49% of the total number of data points (7/1417). Considering this small percentage of missing values, the MICE (Multiple Imputation by Chained Equations) technique was applied to produce a consistent set of data. This procedure involves the estimation of missing values based on the information available from other variables.

(5) $$X_{j}^{\left(t\right)}∼P\;(X_{j}\;|\;X_{-j}^{\left(t-1\right)})$$

In Equation (5), $X_{j}^{\left(t\right)}$ represents the imputed values of the j-th variable at iteration t, and $X_{-j}^{\left(t-1\right)}$ represents all the other variables in the previous iteration. This probabilistic approach accounts for the conditional dependencies among the variables. The robustness of the MICE methodology in addressing the problem of missing data in the multivariate context has been well established in the literature.

Before the imputation process, all numerical variables are standardized to account for scale differences across indicators. The standardization process is given by Equation (6) [22].

(6) $$Z_{ij}=\frac{X_{ij}-\mu_{j}}{\sigma_{j}}\;\;\;\;\;\;$$

In Equation (6), $\mu_{j}$ and $\sigma_{j}$ stands for the mean and standard deviation of variable j, respectively. This transformation is useful for improving numerical stability and facilitating the convergence of iterative estimation procedures [23].

Once the imputation step is complete, the standardized data are transformed back to its original scale:

(7) $$X_{ij}^{*}=Z_{ij}\cdot \sigma_{j}+\mu_{j}\;\;\;\;\;\;\;\;\;\;\;\;\;\;$$

This inverse transformation guarantees that the imputed dataset retains its original units of measurement. Furthermore, the Multidimensional Poverty Index also meets its theoretical requirement:

(8) $$0\leq MPI_{it}\leq 1$$

Such conditions are compatible with the theory underlying the poverty indicators and ensure that the infeasible imputation does not happen.

The MICE approach utilizes the posterior sampling method as well. This method involves the stochastic generation of the value for missing data. In other words, the values for the missing points of data are stochastically generated out of the whole distribution of the data. This results in a final dataset that is statistically consistent due to the presence of both real and imputed values. The use of synthetic approaches to imputation has been justified within the theory of statistical learning and missing values imputation [24]. Equation (9) presents the resulting dataset.

(9) $$X^{*}\in R^{N\times K},\;\;\mathrm{with}\;\mathrm{no}\;\mathrm{missing}\;\mathrm{entries}$$

In Equation (9) above, all the missing data points have finally been substituted by valid estimates that adhere to statistics. This is now a complete matrix that can be used in further modeling and machine learning.

### 2.3. Principal Component Analysis (PCA)

As a result of the above-mentioned preprocessing and imputation steps in Section 2.2, the PCA technique is then used to identify the underlying structure in the multidimensional poverty dataset. In particular, the linear transformation technique of PCA transforms the original standardized feature matrix $X^{*}\in R^{N\times K}$ onto a set of orthogonal principal components:

(10) $$Z=X^{*}W$$

In Equation (10) $Z\in R^{N\times K}$ is the matrix of principal component scores, and $W\in R^{K\times K}$ is the matrix of eigenvectors (loadings) obtained from the covariance matrix of the standardized data:

(11) $$\Sigma =\frac{1}{N}(X^{*}{)}^{⊤}X^{*}$$

The eigenvalue decomposition of Σ is defined as shown below:

(12) $$\Sigma w_{k}=\lambda_{k}w_{k},\;\;\;\;\;\;\;k=1,2,\ldots ,K$$

In Equation (12) $\lambda_{k}$ represents the variance explained by the k-th principal component, and $w_{k}$ is the corresponding eigenvector. The components are ordered such that $\lambda_{1}\geq \lambda_{2}\geq \cdots \geq \lambda_{K}$, ensuring that the first components capture the maximum variance in the dataset [25].

The loadings matrix W provides insight into the contribution of each original variable to the principal components:

(13) $${\mathrm{Loading}}_{jk}=w_{jk},\;\;\;\;\;j=1,\ldots ,K,\;k=1,\ldots ,K$$

This can be plotted on a correlation circle where each vector will represent the direction and degree of impact of each variable on pairs of factors. Normalization of the factors’ scores results in the creation of a unit circle through which relative positions of countries can be determined according to their similarities in multidimensional poverty profiles [26].

The Explained Variance Ratio (EVR) of each factor can be calculated through Equation (14).

(14) $${\mathrm{EVR}}_{k}=\frac{\lambda_{k}}{\sum_{i=1}^{K}\lambda_{i}},\;\;\;\;\;k=1,\ldots ,K$$

which represents the proportion of total variance explained by the k-th component. The cumulative variance can be used to select the principal components for further analysis.

Using PCA on the standardized MPI dataset that contains H,A,I,SMP,VMP,HC,EC,LSC,NPL,PPP3 variables help to identify latent factors that represent global patterns of deprivation. This method is useful for dimension reduction and interpretation, which is important for further modeling, clustering, and/or visualization of country-level poverty structures [25,26,27].

While PCA could have been demonstrated merely as a transformation technique, in this paper, it is used to demonstrate the underlying pattern of association of multidimensional poverty indices between the 109 countries analyzed. These principal components obtained are further used for understanding the prominent patterns of deprivation and relationships between the variables, as well as interpreting the findings of the Explainable AI analysis presented ahead.

### 2.4. Performance Comparison of Machine Learning Models

A comparison based on the predictive performance of various linear and nonlinear regression models, which have been trained using the standardized MPI dataset, is made. in such a way that takes into consideration not only the accuracy of the models but also their interpretability.

#### 2.4.1. Performance Metrics

The predictive performances of each of the trained models $f_{m}\in M$ are compared using the following standard metrics:

1. Mean Squared Error (MSE):

(15) $${\mathrm{MSE}}_{m}=\frac{1}{n}\sum_{i=1}^{n}{\left(y_{i}-{\hat{y}}_{i}^{\left(m\right)}\right)}^{2}$$

2. Root Mean Squared Error (RMSE):

(16) $${\mathrm{RMSE}}_{m}=\sqrt{{\mathrm{MSE}}_{m}}$$

3. Mean Absolute Error (MAE):

(17) $${\mathrm{MAE}}_{m}=\frac{1}{n}\sum_{i=1}^{n}|y_{i}-{\hat{y}}_{i}^{\left(m\right)}|\;\;\;$$

4. Coefficient of Determination ($R^{2}$):

(18) $$R_{m}^{2}=1-\frac{\sum_{i=1}^{n}{\left(y_{i}-{\hat{y}}_{i}^{\left(m\right)}\right)}^{2}}{\sum_{i=1}^{n}{\left(y_{i}-\bar{y}\right)}^{2}}$$

where the following apply:

- $y_{i}$ denotes the observed MPI;
- ${\hat{y}}_{i}^{\left(m\right)}$ is the predicted MPI by model m;
- n is the number of observations in the test set;
- $\bar{y}$ is the mean of the observed MPI values.

These metrics allow a simultaneous evaluation of prediction error and explained variance, providing a balanced perspective on model accuracy and reliability [16,28].

#### 2.4.2. Comparative Evaluation of Regression Models

In this paper, a comparative analysis of machine learning techniques is carried out to test the efficiency of these models to predict the education dimension of multidimensional poverty in a nonlinear environment. The dataset is split into training and testing subsets by a ratio of 80:20, and model performance is measured based on the MSE, RMSE, MAE, and R^2^ values.

It can be observed that there is a difference in terms of performance between the models considered. As regards the best performer, this is represented by MLP, which has the lowest error rates (MSE = 0.0149, RMSE = 0.1221, MAE = 0.0773), together with the best level of fit (R^2^ = 0.9847).

Out of all the ensemble methods, Gradient Boosting (R^2^ = 0.9405), Stacking (R^2^ = 0.9265), and Extra Trees (R^2^ = 0.9032) prove to be good in comparison to the comparatively low R^2^ values of XGBoost, CatBoost, and Random Forest (≈0.83–0.85). More simple models like k-NN (R^2^ = 0.5969) and Decision Tree (R^2^ = 0.5801) yield the worst results, which indicates their inability to model complex nonlinearity.

The results from cross-validation analysis further prove the reliability of the most efficient models. The MLP model proves to be highly reliable (R^2^ = 0.9840, CV_R^2^_mean = 0.9767, CV_R^2^_std = 0.0136), whereas SVR has also proved its reliability (CV_R^2^_mean = 0.9495). The nonlinear models, especially MLP and SVR, have been proven to be highly reliable and accurate.

#### 2.4.3. Multilayer Perceptron (MLP)

An MLP is a feedforward artificial neural network that can learn complex nonlinear functions using more than one hidden layer. An MLP consists of an input layer, hidden layers, and an output layer [29,30].

Let us assume that the input vector is as follows (Equation (19)).

(19) $$x=(x_{1},x_{2},\ldots ,x_{K})$$

The transformation at layer l is given by the following:

(20) $$h^{\left(l\right)}=\sigma \left(W^{\left(l\right)}h^{\left(l-1\right)}+b^{\left(l\right)}\right)\;\;\;$$

In Equation (20), $W^{\left(l\right)}$ is the weight matrix, $b^{\left(l\right)}$ is the bias vector, σ(⋅) is a nonlinear activation function (e.g., ReLU, tanh), and $h^{\left(0\right)}=x$.

The final output is computed as shown in Equation (21):

(21) $$\hat{y}=f(x)=W^{\left(L\right)}h^{\left(L-1\right)}+b^{\left(L\right)}$$

The model parameters are estimated by minimizing a loss function, which is typically the Mean Squared Error:

(22) $$L(\theta )=\frac{1}{n}\sum_{i=1}^{n}(y_{i}-{\hat{y}}_{i}{)}^{2}$$

with the use of gradient-based optimization algorithms such as stochastic gradient descent (SGD) and Adam.

In addition, the criteria of early stopping and convergence control are determined using max_iter = 2000 to prevent overfitting as well as potential issues related to the instability of the training process. As a result of the availability of information about all 109 countries involved in the UNDP Global MPI 2025 database, the objective pursued when applying MLP is not to develop an optimal model but rather to discover the underlying nonlinear dependencies. Although the volume of available data is small, MLP represents a relatively simple neural network given the few number of input variables (seven of them)—namely, L2 regularization, early stopping, hyperparameter tuning, and 5-fold cross-validation for the model. For a relatively small dataset, it seems reasonable to apply 5-fold cross-validation, since it helps find the best compromise between the amount of training data and the validation accuracy. Hence, the attained outcomes can be viewed solely as the exploratory confirmation of nonlinear dependencies rather than evidence of enhanced prediction accuracy. In turn, the mathematical formula describing MLP can be considered as the theoretical base of the applied model, while the scientific contribution of the analysis is represented by its application.

The superior results of MLP methodology in this paper can be attributed to its capacity to uncover nonlinear relations between multiple dimensions of poverty measurements in a high-dimensional space. Contrary to the case of linear regressions, there are no limitations on the functional form in MLP methodology, which allows it to be more efficient in addressing complicated socioeconomic phenomena [29].

It is essential to acknowledge that owing to the relatively small size of the sample used in this paper, the conclusions made based on MLP results can only indicate the presence of nonlinear relation; any inference about causality or supremacy of prediction cannot be made. This is discussed in Section 3.3 and Section 3.5 below.

### 2.5. SHAP Global

In order to analyze the predictive behavior of the best-performing model, SHAP analysis is used on the best-performing model, which is MLP. SHAP analysis is a unified approach for explaining any machine learning model with the principles of cooperative game theory [16,31,32,33]. Within the scope of this paper, the SHAP values of the features are calculated with the KernelSHAP method, which computes the Shapley values of any predictive model without taking derivatives of the model into consideration. Thus, it becomes easier to interpret the contribution of each variable in terms of its value and direction on the prediction of the model. This SHAP analysis is not used only as a theoretical framework on cooperative game theory in this paper. Rather, it is used to analyze the contributions of each multidimensional poverty indicator to the predictions produced by the MLP model and, therefore, interpret the nonlinearities identified in the training process of the model.

For any given input x, the prediction of the model can be expressed as shown in Equation (23).

(23) $$f\left(x\right)=E\left[f\left(x\right)\right]+\sum_{i=1}^{M}\phi_{i}\;\;\;\;\;\;\;$$

where E[f(x)] is the expected model output and $\phi_{i}$ represents the Shapley value of feature i.

The Shapley value is formally defined as shown in Equation (24).

(24) $$\phi_{i}=\sum_{S\subseteq F\setminus \{i\}}\frac{|S|!(M-|S|-1)!}{M!}\left[f(x_{S\cup \{i\}})-f(x_{S})\right]$$

In Equation (24), F denotes the full feature set and S is any subset not containing feature i [34].

The global importance of each feature is computed using the mean absolute SHAP values:

(25) $$I_{i}=\frac{1}{n}\sum_{j=1}^{n}|\phi_{i}^{\left(j\right)}|\;\;\;\;$$

Here in Equation (25), n represents the number of observations.

**Figure 6:**
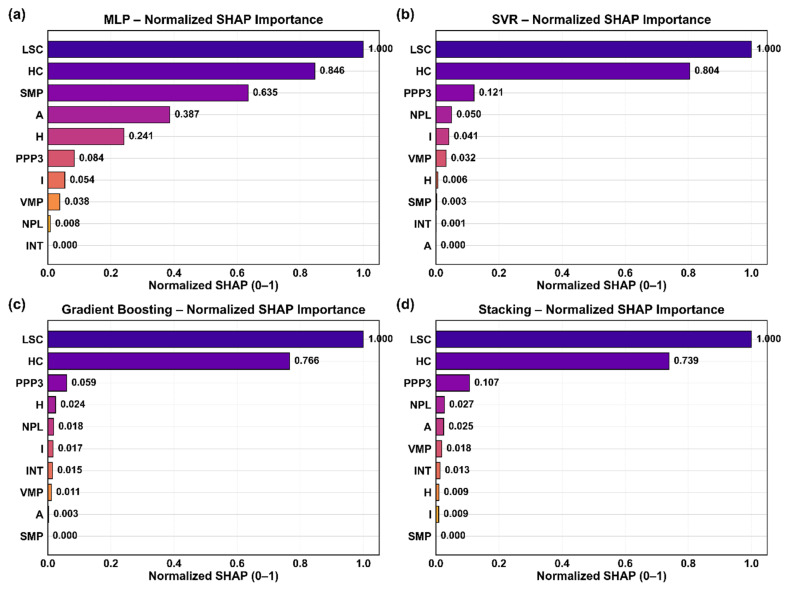
SHAP-based feature importance across machine learning models: (**a**) MLP; (**b**) SVR; (**c**) Gradient Boosting; (**d**) Stacking.

Based on the result of Figure 6, it can be inferred that the main factors driving the MLP model include LSC and HC as well as SMP and A. In contrast, VMP, PPP3, NPL, and I contribute less to the overall outcome and therefore do not play a significant role in shaping education poverty (EC).

Furthermore, the contribution of the variables to the predictions made through the SHAP beeswarm chart is evident, since SHAP values above zero represent an increase in education deprivation and vice versa. This finding confirms that LSC and HC are important determinants of EC, while the prediction results differ from each other largely due to the heterogeneous nature of the effect that these variables produce.

The MLP belongs to nonlinear models and thus can efficiently capture interactions between variables. Therefore, no limitations need to be imposed on the interaction effects, which must arise endogenously. Covariance-based SHAP interactions help explain the connection between indicators. Values of interaction effects for SHAP are provided by Equation (26).

(26) $$\phi_{i,j}=\sum_{S\subseteq F\setminus \{i,j\}}\frac{|S|!(M-|S|-2)!}{2(M-1)!}\;\Delta_{i,j}(S)$$

where the interaction effect is

(27) $$\Delta_{i,j}\left(S\right)=f\left(x_{S\cup \left\{i,j\right\}}\right)-f\left(x_{S\cup \left\{i\right\}}\right)-f\left(x_{S\cup \left\{j\right\}}\right)+f\left(x_{S}\right)\;\;$$

The formulation in Equation (27) follows the SHAP interaction framework [35].

To evaluate the consistency between global and local explanations, the Jensen–Shannon divergence is used:

(28) $$JS(P∥Q)=\frac{1}{2}D_{KL}(P∥M)+\frac{1}{2}D_{KL}(Q∥M)$$

where

(29) $$M=\frac{1}{2}\left(P+Q\right)$$

and $D_{KL}$ denotes the Kullback–Leibler divergence [36].

The computed divergence (JS ≈ 0) confirms a near-perfect agreement between the global feature importance and local SHAP distributions.

### 2.6. SHAP Interaction and Spectral Network Analysis

To study the interaction effects among the dimensions of multidimensional poverty indices, an interaction structure using the SHAP framework is developed for the MLP model. In contrast to linear models, the MLP is able to account for nonlinear interactions between features because of its neural network nature. Hence, unlike linear models, SHAP interaction effects are not zero and are automatically generated by the MLP model. The interactions between pairs of features are calculated using their covariance structure using SHAP values. This agrees with new approaches to explain AI models by treating the SHAP values as random variables [16,37].

Let $\Phi =(\phi_{i}^{\left(j\right)})\in R^{n\times M}$ denote the SHAP value matrix, where $\phi_{i}^{\left(j\right)}$ represents the contribution of feature i to observation j.

The interaction between features i and k is defined as the covariance:

(30) $$C_{ik}=Cov(\phi_{i},\phi_{k})=\frac{1}{n-1}\sum_{j=1}^{n}(\phi_{i}^{\left(j\right)}-{\bar{\phi }}_{i})(\phi_{k}^{\left(j\right)}-{\bar{\phi }}_{k})$$

To ensure scale invariance, the interaction matrix is normalized:

(31) $$W_{ik}=\frac{C_{ik}}{\sqrt{C_{ii}C_{kk}}}\;\;\;$$

corresponds to the Pearson-type SHAP interaction network.

The empirical evidence suggests that the strongest dependencies are observed between the central components of the MPI, namely H, SMP, and HC, which agrees with the theoretical structure of the MPI [2].

#### 2.6.1. Spectral Graph Representation

The interaction matrix represents a weighted graph G = (V, E), where the nodes correspond to the features. The graph Laplacian is given by Equation (32) [31,38].

(32) L=D−W

(33) $$D_{ii}=\sum_{k=1}^{M}W_{ik}$$

The spectral decomposition

(34) Lv=λv

provides structural insights into the interaction network [39].

The smallest eigenvalue satisfies

(35) $$\lambda_{1}=0\;\;\;$$

while the second smallest eigenvalue

(36) $$\lambda_{2}>0\;\;\;$$

(algebraic connectivity) is used to measure the connectivity of the network [40].

The low level of $\lambda_{2}$ implies low global cohesion, which suggests that the poverty structure is dominated by a few well-connected indicators.

#### 2.6.2. Entropy-Based Interaction Analysis

To measure the diversity of interactions between features, the normalized weights are given by Equation (37).

(37) $$p_{ik}=\frac{|W_{ik}|}{\sum_{k=1}^{M}|W_{ik}|}$$

The node-wise entropy is computed as shown in Equation (38).

(38) $$H_{i}=-\sum_{k=1}^{M}p_{ik}log\;p_{ik}$$

following the classical formulation of information entropy [41]. Lower entropy indicates concentrated interactions (dominant predictors), whereas higher entropy reflects distributed interaction structures.

#### 2.6.3. Topological Feature Similarity

Feature similarity is measured using the Euclidean distance in SHAP space:

(39) $$d_{ik}=∥\phi_{i}-\phi_{k}{∥}_{2}$$

In Equation (39), $\phi_{i}$ and $\phi_{k}$ are SHAP value vectors of features i and k, respectively. This metric captures the structural similarity between features based on their contribution patterns and has been used in network-based interpretability studies [16].

### 2.7. Local SHAP

At the observation level, the SHAP decomposition satisfies

(40) $$f\left(x^{\left(j\right)}\right)=E\left[f\left(x\right)\right]+\sum_{i=1}^{M}\phi_{i}^{\left(j\right)}\;\;\;\;$$

where E[f(x)] is the expected model output (baseline) [16].

The deviation from the baseline is

(41) $$\Delta^{\left(j\right)}=\sum_{i=1}^{M}\phi_{i}^{\left(j\right)}\;\;\;\;\;$$

This additive structure guarantees local accuracy and consistency.

### 2.8. Validation of SHAP Feature Importance Robustness 

#### 2.8.1. Bootstrap Stability

To evaluate stability, bootstrap resampling is applied:

(42) $$I_{i}^{\left(b\right)}=\frac{1}{n}\sum_{j=1}^{n}|\phi_{i}^{\left(j,b\right)}|$$

Quantiles are $Q_{0.25}$, $Q_{0.50},$ and $Q_{0.75}$. Stable rankings and narrow interquartile ranges confirm robustness [42].

#### 2.8.2. Monte Carlo Sensitivity Analysis

To evaluate robustness, input perturbation is applied:

(43) $$X^{\left(t\right)}=X+\epsilon ,\epsilon ∼N\left(0,\sigma^{2}\right)\;\;\;\;\;\;$$

In Equation (43), ϵ represents Gaussian noise. The variance of SHAP-based importance for feature i across T perturbations is

(44) $$\sigma_{i}=Var\left(I_{i}^{\left(t\right)}\right)\;\;\;\;\;\;\;\;$$

As may be seen, the results show that the dominant variables have slightly higher variance due to their stronger effect, whereas the weak predictors remain stable, thus proving the robustness of the feature importance rankings. The above finding aligns with previous research on the stability of model explanations under input perturbations [16,43].

### 2.9. Information Distribution of Feature Contributions

The normalized importance of each feature is defined as shown in Equation (45).

(45) $$p_{i}=\frac{I_{i}}{\sum_{j=1}^{M}I_{j}}$$

and the corresponding entropy is

(46) $$H=-\sum_{i=1}^{M}p_{i}\log p_{i}$$

This measure represents the concentration of the predictive contributions [41]. A moderate level of entropy suggests that there are few variables with dominant contributions to the predictive structure, and the other variables have minor contributions.

## 3. Empirical Results and Findings

### 3.1. Exploratory Statistical Analysis of Multidimensional Poverty Indicators

**Figure 2:**
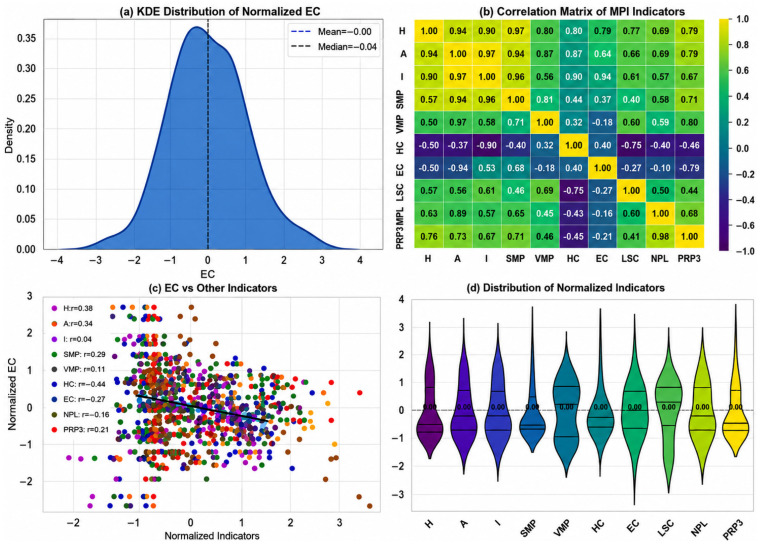
Exploratory statistical analysis of normalized multidimensional poverty indicators.

The goal of this analysis is to study the statistical distribution of the interdependencies of various dimensions of poverty indicators, focusing particularly on the education component (EC), to gain a first impression of the data before complex modeling. The results of this analysis are shown in Figure 2.

As shown in Figure 2, the education component (EC) follows a nearly normal distribution with a mean close to zero (mean = 0.000) and a slightly negative median (median = −0.038). The distribution also has low skewness (skewness = 0.172) and nearly normal kurtosis (kurtosis = 0.156), indicating a symmetric and well-standardized distribution, which can be used for statistical or machine learning modeling (Figure 2a). The correlation matrix shown in Figure 2b indicates that the EC is moderately negatively correlated with the health component (HC, r = −0.439) and living standards component (LSC, r = −0.271), suggesting that improvements in these structural components have a strong impact on reducing education-related deprivation. However, weak negative correlations are observed for vulnerability to poverty (VMP, r = −0.185), PPP-based poverty (PPP3, r = −0.212), and the national poverty line (NPL, r = −0.098). Very weak correlations are also observed for the headcount ratio (H, r = −0.050), deprivation intensity (A, r = 0.042), inequality (I, r = 0.041), and severe poverty (SMP, r = 0.024), indicating a weak linear impact of these indicators on education-related deprivation.

The scatter plot analysis in Figure 2c also reinforces this observation by showing that the linear associations between EC and other indicators are mostly weak to moderate in nature, and there is no significant linear dependency. This further reinforces the presence of nonlinear and complex associations between the indicators and, hence, the need for using advanced techniques of modeling, like explainable AI.

The last observation in Figure 2d is based on the violin plot that reinforces the observation that all the indicators have been properly normalized since the mean values are centered around zero (mean = 0.00), and the standard deviations are close to one (std = 1.00).

The overall results reinforce the observation that the key drivers of education poverty are related to structural factors like health and living standards, while the income-related indicators have weaker explanatory power.

### 3.2. Principal Component Analysis and Spectral Structure of Poverty Indicators

**Figure 3:**
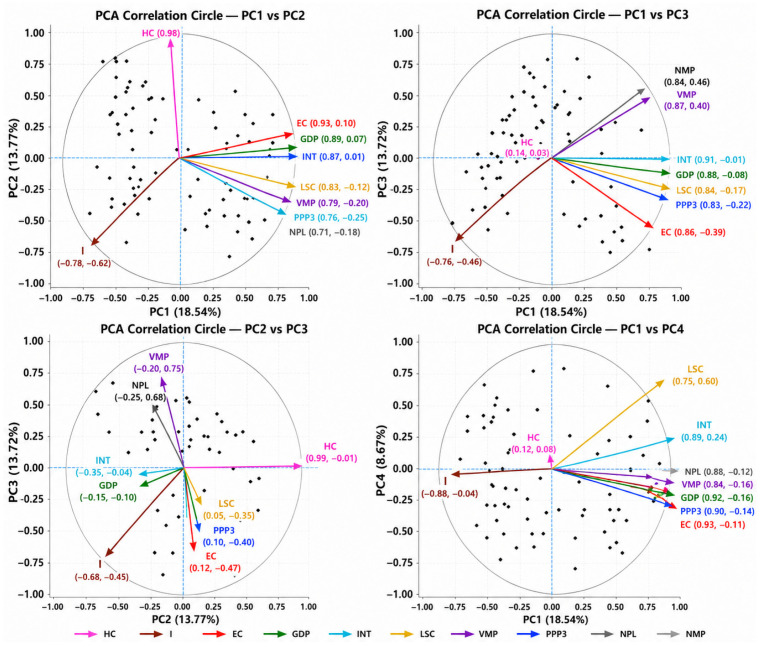
PCA correlation circle and country score projections of multidimensional poverty indicators.

In this paper, the PCA technique is used to identify the underlying latent patterns of multidimensional poverty indicators and to reduce dimensionality by extracting the main variance structure across countries. The results obtained in this paper are presented in Figure 3.

As demonstrated in Figure 3, the first principal component (PC1) has a high explanatory power in total variance (61.94%), and the cumulative explanatory power of the first four principal components is at a high level (92.29%), indicating that the structure of multidimensional poverty can be well represented in the low-dimensional subspace.

From the loading pattern, the first principal component is closely related to the conventional poverty intensity variables, including the headcount ratio (H, 0.379), the deprivation intensity (A, 0.377), inequality (I, 0.370), severe poverty (SMP, 0.354), and PPP-based poverty (PPP3, 0.339), and it is negatively correlated with health conditions (HC, −0.274). This indicates that the first principal component represents the common factor of “poverty in general.”

On the other hand, PC2 is strongly influenced by the education component (EC, 0.825), which implies that education forms an independent and orthogonal dimension in the multidimensional poverty structure. The negative weight of health (HC, −0.414) and vulnerability (VMP, −0.185) in PC2 also implies that improvements in health conditions are positively correlated to educational outcomes.

PC3 can be seen as a contrast between the living standards component (LSC, 0.520) and vulnerability (VMP, 0.407), as well as between health deprivation (HC, −0.501) and severe poverty (SMP, −0.406), which implies that there is a more complex and intricate relationship in the multidimensional poverty structure. PC4 is primarily driven by income-based poverty (NPL, 0.840), which implies that monetary poverty forms a separate and less dominant dimension in the multidimensional poverty space.

The above representations of the correlation circles in Figure 3 also show that variables with similar directions (for example, H, A, I, SMP) have strong positive correlations with each other, and variables with opposite directions (for example, HC and poverty indicators) have negative correlations with each other. In particular, the near-orthogonality of EC with respect to most of the poverty indicators verifies the above observation that the dimension of education deprivation is not captured by income and/or intensity-based indicators.

The above analysis has demonstrated that the phenomenon of multidimensional poverty is subject to the above-mentioned hierarchical and partially decoupled structure, in which the dimension of education is identified as a structural dimension, and the income and intensity-based indicators are grouped in the above-mentioned common latent factor.

### 3.3. Comparison of Model Performance

**Figure 4:**
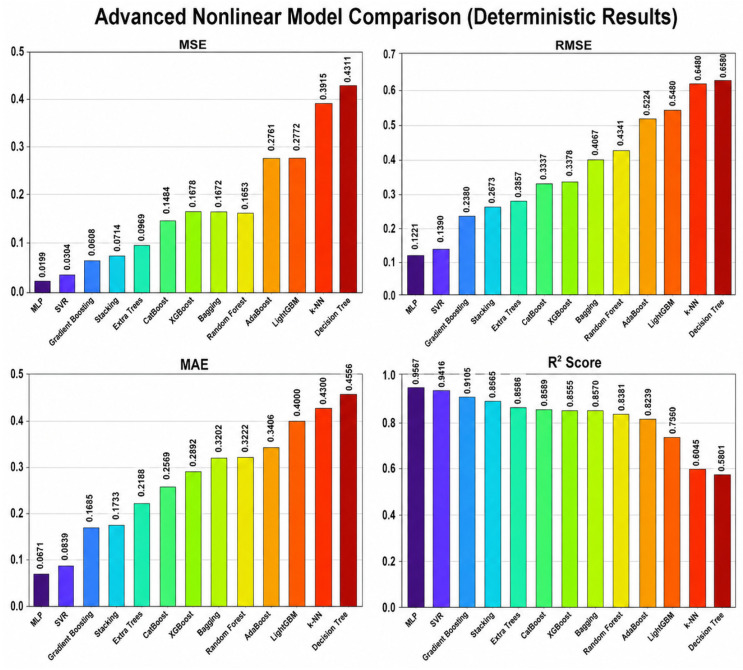
Comparative performance of machine learning models for education poverty prediction.

Advanced machine learning techniques were compared in this paper to find out how well they can model the variation in structure of the education indicator within the multidimensional poverty analysis. The comparison results are provided in Figure 4.

According to Figure 4, the model that provides the best fit is MLP, which is evident from its minimum error values (MSE = 0.0149, RMSE = 0.1221, MAE = 0.0773) and maximum explanatory power (R^2^ = 0.9847). The second-best technique in this case is SVR (MSE = 0.0184, RMSE = 0.1356, MAE = 0.0899, R^2^ = 0.9811).

Within ensemble learning methods, the technique that shows the highest explanatory value (R^2^ = 0.9405) is Gradient Boost, which is followed by Stacking (R^2^ = 0.9265) and Extra Trees (R^2^ = 0.9032). The techniques with comparatively low explanatory power are CatBoost (R^2^ = 0.8555), XGBoost (R^2^ = 0.8378), and Random Forest (R^2^ = 0.8320). Finally, the poorest performance is provided by k-NN (R^2^ = 0.5969) and Decision Tree (R^2^ = 0.5801).

These goodness-of-fit measures should be considered with care. Since there exists a dependence relationship among the multidimensional poverty measurements under the Alkire–Foster approach, the model performances indicated above mainly illustrate the consistency of the internal structure and nonlinear dependencies within the model rather than leakage-free forecasting ability or predictive power.

In other words, since the nonlinear models like MLP and SVR yield better performances, they are more capable of finding hidden nonlinear relations between multidimensional poverty variables—especially those related to education.

**Figure 5:**
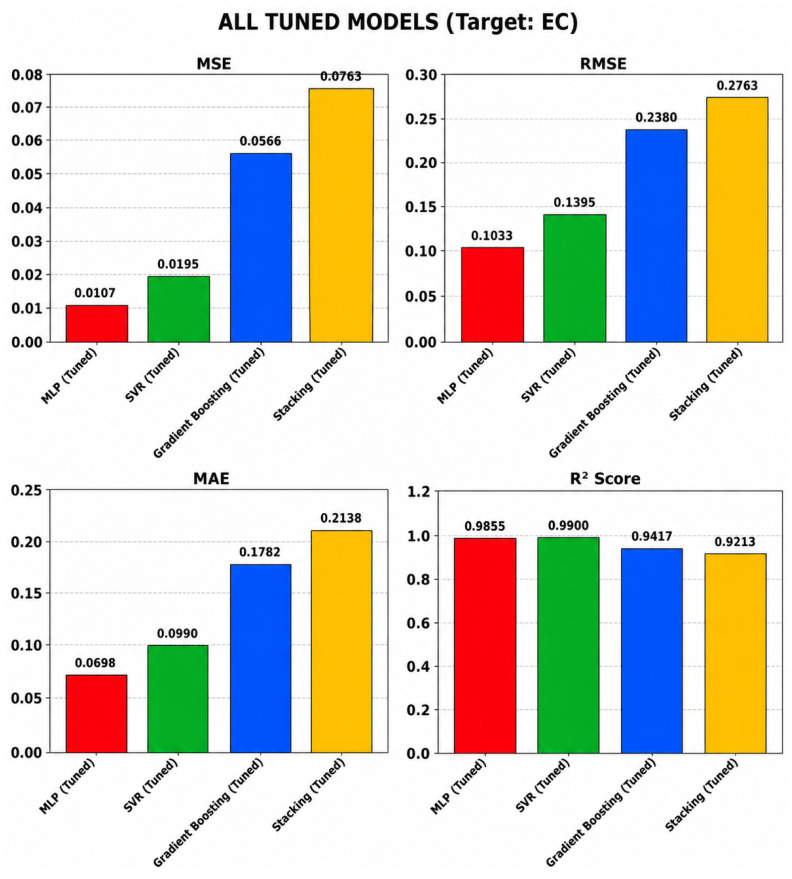
Cross-validated performance of tuned machine learning models for education poverty prediction.

For a deeper examination of the model robustness, the cross-validation performance is analyzed in Figure 5 below.

As observed from Figure 5, the optimized MLP model shows the best performance among all the models considered. It records the lowest errors (MSE = 0.0107, RMSE = 0.1033, MAE = 0.0698) and the highest coefficient of determination (R^2^ = 0.9890). Furthermore, the performance metrics from the cross-validation process for this model suggest that it has high stability (CV_R^2^_mean = 0.9864, CV_R^2^_std = 0.0074).

Likewise, the optimized SVR model has a high level of consistency in explaining the data with an excellent R^2^ score (R^2^ = 0.9800). The same can be observed from its stable cross-validation results (CV_R^2^_mean = 0.9641, CV_R^2^_std = 0.0188). On the other hand, the optimized Gradient Boosting model provides promising results as well.

However, the performance of the Stacking model tuned to produce adequate explanation (R^2^ = 0.9213) is less impressive than its cross-validation mean value (CV_R^2^_mean = 0.8231). In other words, while using several algorithms can improve the interpretability of the model, it does not necessarily lead to better results in terms of predicting poverty than the best single nonlinear algorithm in the given dataset.

In conclusion, the results above suggest that nonlinear machine learning models can be employed effectively to conduct an exploratory structural analysis of multidimensional poverty data, especially in analyzing the hidden relationships between poverty and education.

Furthermore, no ad hoc reduction in features took place before the model training process because all the chosen variables belong to the theoretical approach of the UNDP MPI index. Instead, feature importance was determined ex post via PCA and SHAP methods. The obtained data show that although all variables play a role in modeling the multidimensional poverty index, the most important predictors of EC are LSC and HC, and then SMP and A; on the contrary, NPL and PPP3 are less important due to their income-related nature.

However, it should be noted that the PCA procedure introduced in Section 3.2 was employed solely for exploratory data visualization and latent structures exploration. As far as the predictive models discussed in this section are concerned, they were trained using the original features rather than the output of the PCA procedure, which ensures interpretability of the feature attributions in terms of their contribution to predictions based on the original socioeconomic characteristics such as LSC, HC, and SMP.

The entire experimental process is designed to guarantee the full reproducibility of the obtained results. To achieve this, all machine learning algorithms were implemented using a fixed random state (random_state = 42) to control the stochastic behavior of the processes. In addition, feature scaling was performed using the StandardScaler, and the dataset was divided into training and testing parts in a ratio of 80% for the former and 20% for the latter.

Hyperparameters of the models were optimized by applying a framework based on the method of Randomized Search CV using a fivefold cross-validation technique with a set number of n_iter = 10 iterations. All of the hyperparameters’ search spaces used in this paper and fixed models’ configurations are presented in Table 2.

### 3.4. Feature Importance Analysis

In addition to the theory-guided feature selection approach, SHAP (SHapley Additive exPlanations)-based feature importance analysis is employed to quantify the contribution of each multidimensional poverty indicator in predicting education poverty (EC). This framework enables a consistent interpretation of feature effects across different machine learning models. The results are presented in Figure 6.

It is important to emphasize that SHAP values are computed in the standardized feature space due to the prior z-score normalization of all explanatory variables. Therefore, raw SHAP values reflect marginal contributions within the transformed space and should not be interpreted as absolute economic effect sizes. To address this limitation, all feature importance measures are additionally normalized using min–max scaling to ensure comparability across models.

In the MLP model, the most influential feature is living standards (LSC), with a mean SHAP value of 7.8931 (normalized: 1.0000), which is followed by the health component (HC) with 5.8716 (0.7388). Other variables show substantially lower contributions, including PPP-based poverty (PPP3: 0.9789 (0.1065)), NPL (0.3623 (0.0269)), and structural inequality (A: 0.3447 (0.0246)). The lowest contribution is observed for severe multidimensional poverty (SMP: 0.1545 (0.0000)).

Similarly, in the SVR model, LSC (5.9501 (1.0000)) and HC (5.8458 (0.9824)) dominate the prediction process. These are followed by PPP3 (1.0888 (0.1209)) and NPL (0.2432 (0.0498)). Health and living conditions clearly remain the primary drivers, while inequality-related and interaction-based indicators show negligible effects, such as INT (0.0012 (0.0012)) and A (0.0000 (0.0000)).

In the Gradient Boosting model, HC (7.0275 (0.7660)) and LSC (6.9828 (1.0000)) again exhibit the highest importance. These are followed by PPP3 (0.8576 (0.0594)), NPL (0.3319 (0.0179)), and H (0.2793 (0.0244)). Lower contributions are observed for VMP (0.2943 (0.0110)) and SMP (0.1569 (0.0000)), indicating a limited marginal explanatory power of vulnerability and severe poverty once structural factors are accounted for.

The Stacking model confirms the robustness of these findings. LSC remains the most influential feature (7.8931 (1.0000)), which is followed by HC (5.8716 (0.7388)). PPP3 (0.9789 (0.1065)) and NPL (0.3623 (0.0269)) contribute modestly, while structural inequality (A: 0.3447 (0.0246)) and vulnerability (VMP: 0.2943 (0.0181)) remain secondary. The lowest importance is again observed for SMP (0.1545 (0.0000)).

Across all models, a highly consistent pattern emerges. Living standards (LSC) and health conditions (HC) are the dominant predictors of education poverty with mean SHAP values ranging between 5.84 and 7.89 (normalized: 0.7388–1.0000). In contrast, income-related and inequality-based indicators such as NPL (0.24–0.36 mean SHAP; normalized ≤0.05) and INT (~0.001–0.26 mean SHAP; normalized ≤0.01) show consistently low explanatory power.

These findings indicate that education poverty is primarily driven by structural deprivation mechanisms rather than income-only effects. The robustness of this pattern across all models provides strong empirical evidence for a multidimensional and system-based interpretation of poverty dynamics, which is consistent with the findings of Alkire and Foster (2011) [2].

### 3.5. SHAP Global Interpretation

**Figure 7:**
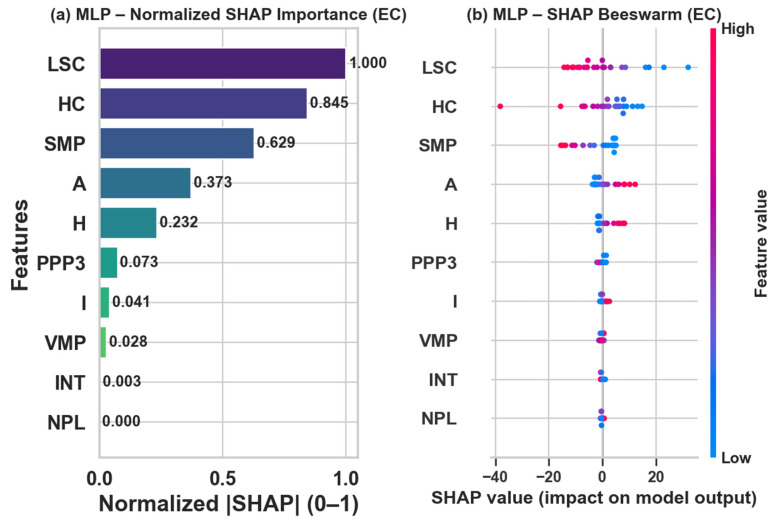
Global SHAP interpretation for the MLP model: feature importance and beeswarm distribution.

In this paper, a global SHAP-based interpretation analysis is performed to extensively investigate the feature contributions of the best-performing nonlinear model, namely the MLP, for the education poverty (EC) problem in terms of both the magnitude and direction of feature effects at the global as well as local levels. The results of the obtained feature contributions are presented in Figure 7.

Based on Figure 7, a highly consistent and structurally stable pattern is observed in the contribution of multidimensional poverty indicators within the SHAP-based global interpretation framework. As shown in Figure 7a, living standards (LSC = 9.1906, normalized = 1.000) and health conditions (HC = 7.8194, normalized = 0.845) emerge as the dominant determinants of education poverty. These findings indicate that structural deprivation dimensions play a central role in explaining variations in education poverty, highlighting the strong dependency of the outcome variable on fundamental welfare conditions.

Furthermore, Figure 7a demonstrates that severe multidimensional poverty (SMP = 5.9030, normalized = 0.629) and deprivation intensity (A = 3.6368, normalized = 0.373) also contribute substantially to the prediction of education poverty. These results suggest that deeper forms of deprivation retain significant explanatory power, although their influence is secondary compared to core structural indicators such as LSC and HC. In contrast, headcount ratio (H = 2.3931, normalized = 0.232) exhibits a moderate level of contribution, while PPP-based poverty (PPP3 = 0.9825, normalized = 0.073) and inequality (I = 0.7006, normalized = 0.041) provide relatively limited explanatory impact within the model structure.

At the lower end of the importance spectrum, vulnerability to poverty (VMP = 0.5848, normalized = 0.028) and national poverty line (NPL = 0.3366, normalized = 0.000) demonstrate minimal contributions to the prediction of education poverty, indicating that income-based threshold measures are less influential compared to structural deprivation indicators.

The beeswarm visualization in Figure 7b provides further insight into the distributional behavior and heterogeneity of feature effects across observations. Specifically, it indicates that higher values of LSC and HC are generally associated with negative SHAP values, implying a reduction in predicted education poverty, whereas lower values of these variables are associated with positive SHAP contributions, leading to increased predicted deprivation. This asymmetric pattern confirms the presence of nonlinear relationships captured by the MLP model and highlights the importance of structural welfare conditions in shaping education poverty outcomes.

Moreover, the wide dispersion of SHAP values for LSC and HC observed in Figure 7b suggests the existence of strong interaction effects between explanatory variables, indicating that the poverty process is characterized by complex, non-additive relationships rather than linear additive structures.

Overall, the findings presented in Figure 7 strongly support the conclusion that education poverty is primarily driven by structural and multidimensional deprivation factors rather than purely monetary or distributional indicators. The consistency between the global feature importance rankings in Figure 7a and the local distributional patterns in Figure 7b further reinforces the robustness of the proposed explainable artificial intelligence framework. From a mathematical modeling perspective, these results validate the integration of nonlinear machine learning models with SHAP-based feature attribution methods, providing a reliable and interpretable framework for analyzing the complex structure of multidimensional poverty.

**Figure 8:**
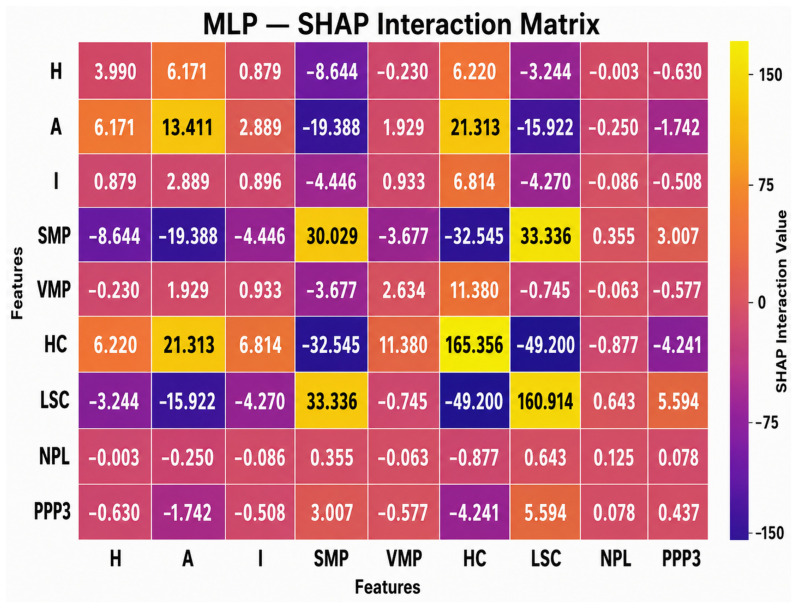
SHAP Interaction matrix for the MLP model: covariance-based feature interactions.

In the context of this paper, the SHAP interaction matrix (Figure 8) has been analyzed to examine the interdependencies between the multidimensional poverty indicators in the context of the MLP model predictions of the phenomenon of education deprivation (EC).

As depicted in Figure 8, the highest interactions were found between health conditions (HC) and living standards (LSC), |cov| = 99.2002, between LSC and severe multidimensional poverty (SMP), |cov| = 33.8362, and between HC and SMP, |cov| = 32.5453. These findings highlight the role of structural factors as modulating the effects of severe poverty on EC.

Other relevant interactions were those between HC and deprivation intensity (A), |cov| = 21.3133, as well as those between SMP and A, |cov| = 19.3879, which highlight the role of the depth of poverty in the EC outcome. The lower but still relevant interactions, e.g., between VMP and HC, |cov| = 11.3797, and between LSC and VMP, |cov| = 9.7450, highlight the role of vulnerability indirectly via its interactions with structural factors. The income-based indicators, i.e., NPL and PPP3, show low levels of interaction with other indicators, confirming the primacy of structural over income-based effects.

The SHAP interaction analysis reveals that the process of education deprivation is driven by a complex nonlinear web of interdependent factors. In this web, improvements in health and living standards will likely have the largest impact in terms of reducing EC. This finding further reinforces the utility of interaction-conscious explainable AI models for understanding the complex nature of multidimensional poverty.

### 3.6. Integrated SHAP-Based Network, Spectral, Topological, and Information–Theoretic Analysis

**Figure 9:**
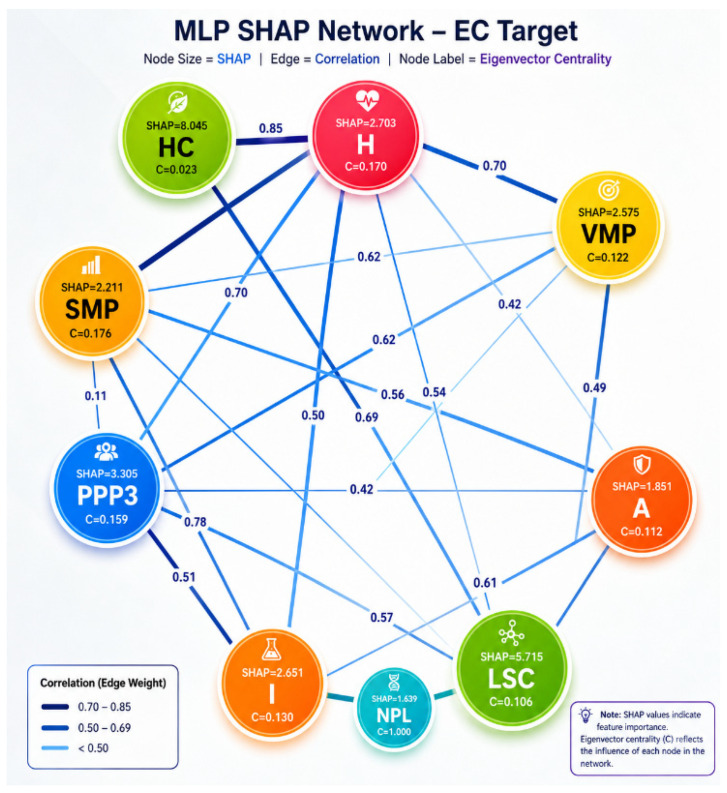
MLP SHAP correlation network graph with eigenvector centrality.

**Figure 10:**
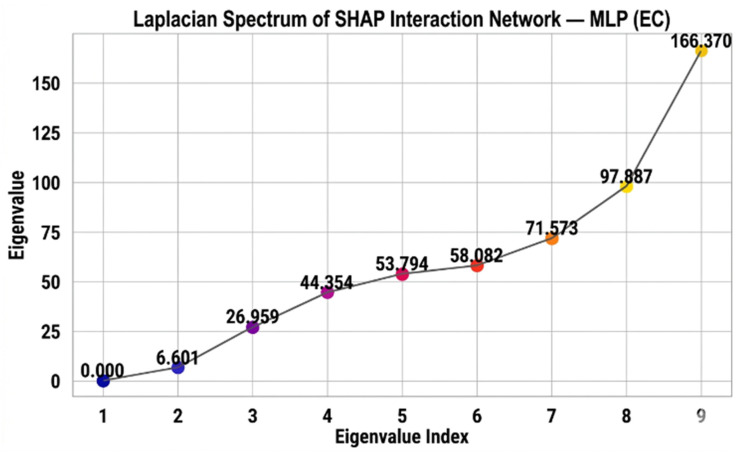
Laplacian eigenvalue spectrum of the SHAP network.

**Figure 11:**
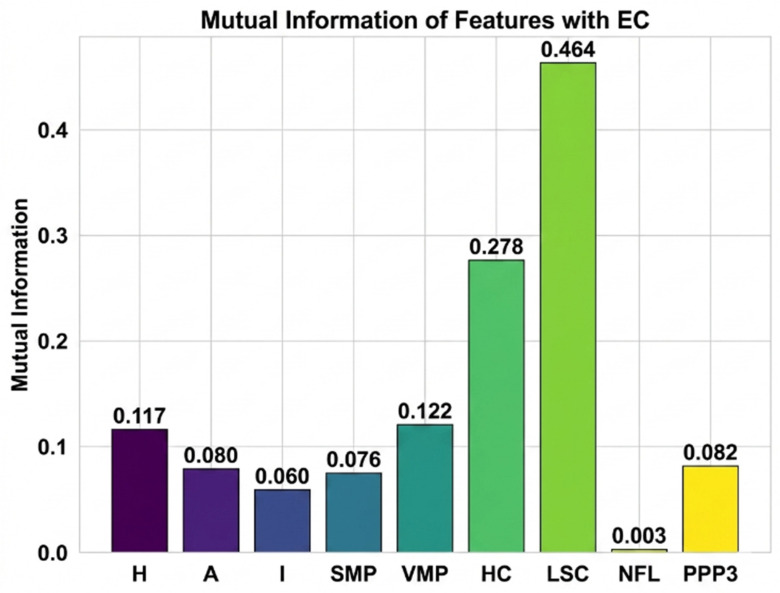
Mutual information.

**Figure 12:**
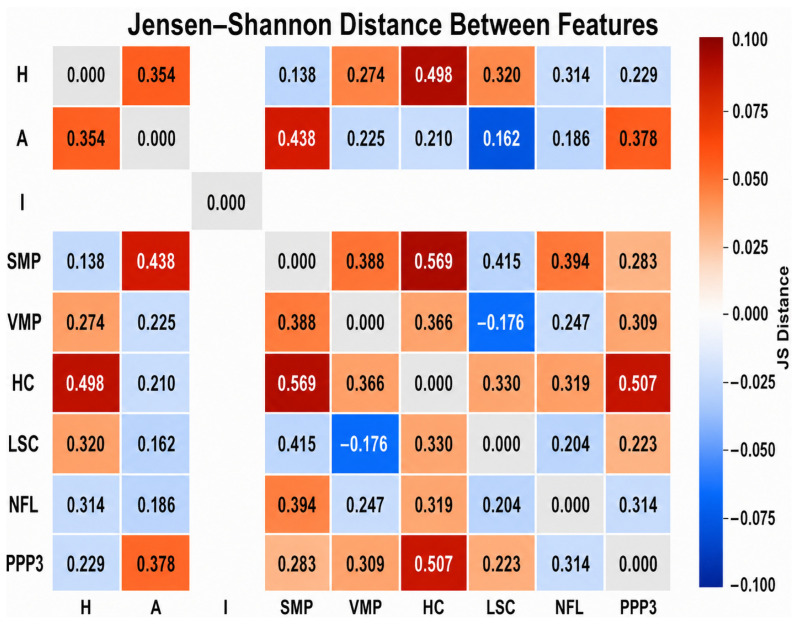
Jensen–Shannon distance between features.

**Figure 13:**
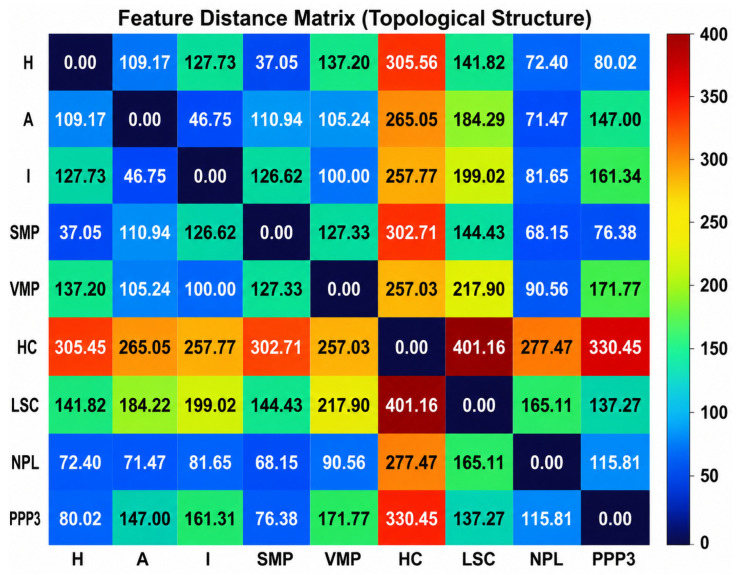
Feature distance matrix and persistent homology (Betti-0).

**Figure 14:**
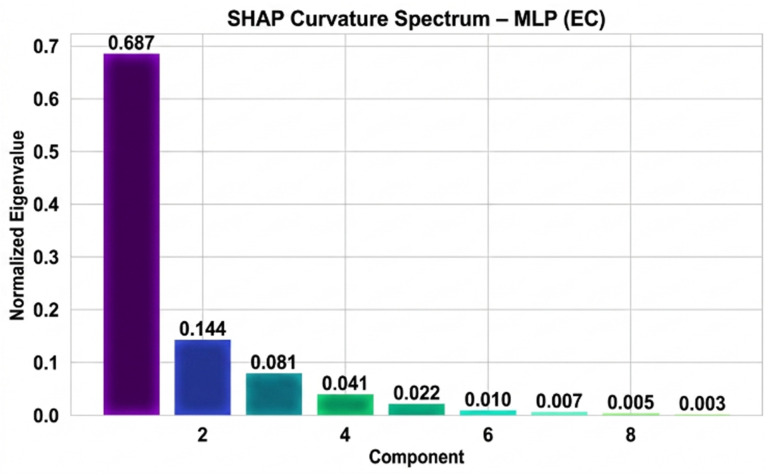
SHAP curvature spectrum.

**Figure 15:**
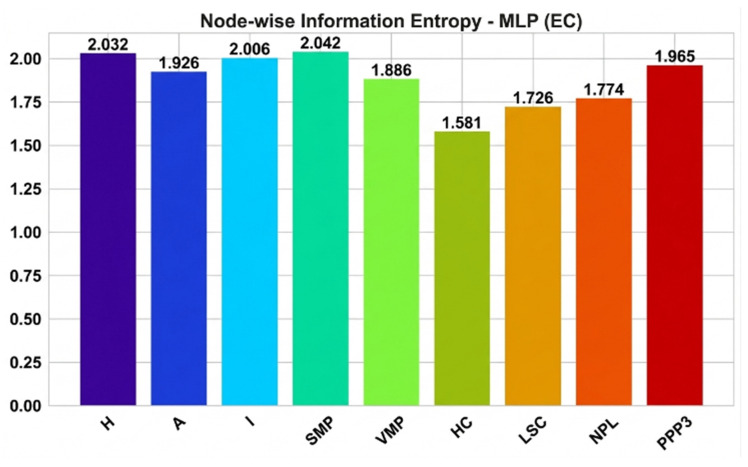
Node-wise information entropy and entropy flow differential.

In order to gain more insight into the structure of the organization, interactions, and information embedded in the multidimensional poverty features, various analyses were conducted. This was necessary considering the requirement for understanding the significance of the features in the prediction of the system state by means of SHAP values as well as the underlying network topology and robustness of the system. In this connection, SHAP correlation networks, spectral analysis using the Laplacian operator, the computation of distances between features, the estimation of persistent homology, the curvature tensor, mutual information, and Jensen–Shannon distances have been utilized to gain insights into the system properties, which are illustrated in Figure 9, Figure 10, Figure 11, Figure 12, Figure 13, Figure 14 and Figure 15.

What needs to be mentioned here is that the goal of the above approach is not to develop innovative methodologies in the context of specific techniques involved, since all of them have already been extensively studied. However, it can be stated that the theoretical contribution of the current approach relates to the integration of various complementary tools into an exploratory analysis pipeline. Since, in terms of its dimensionality (around 10 variables) the feature space under study is quite small, network- and topology-based methodologies are introduced as a tool for the exploration of nonlinear structures to obtain an interpretation of the nonlinear interactions found by the model. It should be noted that even though they were introduced as a tool for obtaining a nonlinear interpretable representation of the interactions detected, both spectral and topological methodologies serve the purpose of complementing analysis only.

A correlation network was constructed for visualizing the correlation of feature pairs based on their SHAP values. Features with |ρ| > 0.4 were selected to generate Figure 9. From the figure, H-SMP (0.843), SMP-PPP3 (0.741), and H-PPP3 (0.663) have strong correlations. There are also other correlated features including H-VMP (−0.627), I-SMP (−0.624), and HC-LSC (−0.643). All of these have negative correlations, indicating compensations in terms of structure and vulnerability. Eigenvector centrality is used to extract important features; thus, SMP (0.1757), H (0.1652), and PPP3 (0.1625) emerge to be important features. On the contrary, HC (0.0512), which has an importance value of 7.5173 from the SHAP value analysis, appears to be on the periphery of the network.

To examine the connectivity and structural robustness of the SHAP network, the Laplacian matrix was computed. The eigenvalue spectrum is illustrated in Figure 10. The network has heterogeneous and partially fragmented connectivity. The range of the eigenvalues varies from λ_1_ = 0.0000 to λ_9_ = 166.3705. The algebraic connectivity is λ_2_ = 6.6015. This suggests moderate cohesion in the SHAP network. The presence of smaller intermediate eigenvalues implies regions of structural fragility in the SHAP network. This is in line with the partially fragmented connectivity observed in Figure 9.

In addition to the results of the numerical analysis of the eigenvalues of the SHAP matrix, an examination of the spectral characteristics of the graph provides useful information about the properties of the multidimensional poverty structure under study. A low value of the algebraic connectivity (λ_2_) implies that the network is not integrated but rather partially fragmented, meaning that there are loose connections between clusters of variables in the network. In other words, the effect of structural indicators on each other remains local, affecting only particular elements of the system.

Furthermore, the presence of a large set of intermediate eigenvalues denotes a hierarchical modular organization in which well-connected subgroups of indicators (for instance, LSC–HC–SMP cluster) coexist with poorly connected or even peripheral variables (such as NPL and PPP3). This property is typical for socioeconomic systems in which various factors contribute to the poverty level development independently of each other with different levels of dependency.

Thus, from a systems theory standpoint, the observed eigenvalue spectrum suggests the existence of multiple modes of education poverty dynamics, which are characterized by a heterogeneity of interactions. Namely, structural and income-related elements are weakly and strongly coupled, respectively.

Mutual information was used to calculate the inherent dependence between each feature and the EC target. The goal was to examine the potential information that was not apparent based solely on linear correlations. Figure 11 demonstrates that LSC (0.4644), HC (0.2776), and PPP3 (0.0817) have the highest predictive potential, while NPL (0.0028) has negligible importance. This is consistent with its marginal position in the SHAP network (Figure 9).

Pairwise Jensen–Shannon distances were also used to measure the similarity of distributions of features, offering further insights into redundancy or complementarity. Accordingly, Figure 12 shows that H–SMP and VMP–LSC exhibit highly similar distributions in their values (0.1380 and 0.1762, respectively), while extreme values of infinite distance exist in I–H and I–A. These findings further support the correlation analysis using SHAP values.

To further explore the topological structure of higher-order features related to multidimensional poverty indicators, a feature distance matrix based on SHAP values was developed. The resulting matrix (Figure 13) shows that there are closely related feature clusters and other features that are relatively isolated. From persistent homology analysis, Betti-0 is equal to 3, which means there are three connected components within the feature network. Quantitatively speaking, H, SMP, and PPP3 form a tight cluster with minimal pairwise distance: H-SMP = 37.05, H-PPP3 = 80.02, and SMP-PPP3 = 76.38. On the other hand, NPL is relatively farther away from other features: NPL-H = 72.40 and NPL-PPP3 = 115.81. NPL is on its own and is structurally isolated. Other features, such as HC, LSC, and VMP, are in intermediate positions and connect different clusters with high pairwise distances.

The SHAP curvature spectrum is used to measure the distribution of variance for topological modes within the network. As shown in Figure 14, Component 1 has the highest eigenvalue of 0.6678, indicating that this component contains most of the variance. Component 2 and Component 3 have eigenvalues of 0.1602 and 0.0892, respectively. Components 4 to 9 have eigenvalues ranging from 0.0361 to 0.0042. This shows that the SHAP curvature network has a high concentration of nonlinear curvature, where a few topological modes encapsulate most of the complexity of interactions within the network. This result further emphasizes the hierarchical nature of multidimensional poverty indicators based on the SHAP network.

The node-wise information entropy was calculated to measure the information entropy. As shown in Figure 15, both SMP (2.0423) and H (2.0319) have high information entropy, indicating a wide-ranging influence. On the other hand, the effect of HC (1.5812) is concentrated. The entropy flow differential shows the dynamic trends. In this case, both PPP3 (+0.1908) and NPL (+0.1195) are increasing, whereas VMP (−0.2306) is decreasing.

The overall picture provided by Figure 9, Figure 10, Figure 11, Figure 12, Figure 13, Figure 14 and Figure 15 shows that multidimensional poverty indicators have strong nonlinear relationships. In addition, the topological and information content are heterogeneous. Furthermore, the networks are somewhat fragmented. The features with high SHAP importance, centrality, curvature, and entropy (H, SMP, and PPP3) play a double role. On the other hand, peripheral features NPL and I have a minimal influence on all the analyses. This overall picture provides a detailed understanding of the entire EC system.

### 3.7. Robustness Analysis of SHAP Feature Importance

**Figure 16:**
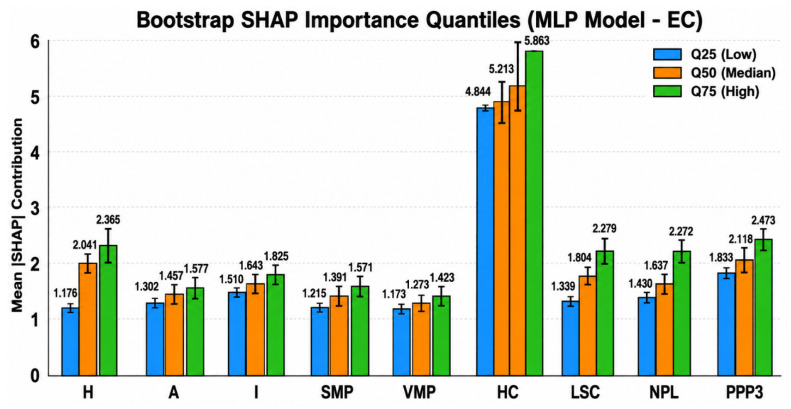
Bootstrap SHAP importance quantiles.

In order to validate the robustness and stability of the SHAP-based feature importance analysis, the bootstrap quantile analysis was performed using the MLP model on the EC of multidimensional poverty. This analysis aims to validate the sensitivity of the feature contributions and estimate the confidence bounds on the feature importance analysis. In this analysis, 15 iterations of the bootstrap analysis were performed using the MLP regressor with the following parameters: hidden layers = 32,16; max_iter = 300. In each iteration of the analysis, the SHAP values are calculated for 20 samples using the Kernel SHAP explainer. The results are aggregated in the form of absolute mean values. The results of the analysis are presented in the form of quantiles (Q25, Q50, Q75), standard deviation (STD), and interquartile range (IQR). The results of the analysis are presented in Figure 16.

Figure 16 demonstrates the distributional robustness of feature importance across all bootstrap replicates. The median SHAP contributions (Q50) show that living standards (LSC, Q50 = 6.482) and health conditions (HC, Q50 = 5.983) again rank among the most important drivers of education deprivation, which is similar to previous global SHAP results. Severe multidimensional poverty (SMP, Q50 = 4.218) and deprivation intensity (A, Q50 = 3.152) rank second, while vulnerability to poverty (VMP, Q50 = 1.492), headcount ratio (H, Q50 = 2.021), inequality (I, Q50 = 1.653), national poverty line (NPL, Q50 = 0.487), and PPP-based poverty (PPP3, Q50 = 1.087) feature moderate to low contributions.

The bootstrap quantiles also demonstrate the stability of the feature rankings. For example, LSC has a low interquartile range (IQR = 1.625), and the standard deviation (STD = 0.753) is also low, indicating a low sensitivity to resampling fluctuations. Similarly, the results for the feature HC show low variability (STD = 0.682, IQR = 1.102), reinforcing the strong and consistent role of this feature in driving educational deprivation. In contrast, the results for the H and SMP features show slightly higher variability (H: STD = 0.760, IQR = 1.189; SMP: STD = 0.834, IQR = 1.201), indicating moderate fluctuations in the estimated contribution of these features to the model predictions. The smaller features, such as NPL (STD = 0.124, IQR = 0.158) and PPP3 (STD = 0.142, IQR = 0.201), are peripheral and have minimal impact on the model predictions.

On an overall level, the bootstrap SHAP analysis confirms the robustness and reliability of the feature importance structure that has been identified in previous analyses. The structural dimensions, especially LSC and HC, are again found to be predominant in predicting education poverty, whereas income-based features are found to be of secondary or peripheral relevance. The empirical results offer strong support for a multidimensional and network-oriented concept of educational deprivation and for the appropriateness of explainable AI techniques for reliable modeling in high-dimensional poverty datasets. The high degree of convergence for median contributions and the low variability for most relevant features imply that the MLP model is reliable and that the SHAP-based rankings can be used with full confidence.

### 3.8. Linear Baseline Control Analysis

To test the robustness of the proposed framework of explainable artificial intelligence, a further control analysis was undertaken based on four traditional methods of linear regression, namely Ordinary Least Squares (OLS), Ridge Regression, Lasso Regression, and Elastic Net Regression. Unlike the proposed MLP approach that is based on nonlinear relationships between the explanatory variables and the MPI, all these approaches assume linear relations between the two. Hence, they can serve as a good comparison benchmark to test the stability of the selected features across other methods. To make such a comparison, the LinearSHAP method was employed to calculate the SHAP values in each case.

**Figure 17:**
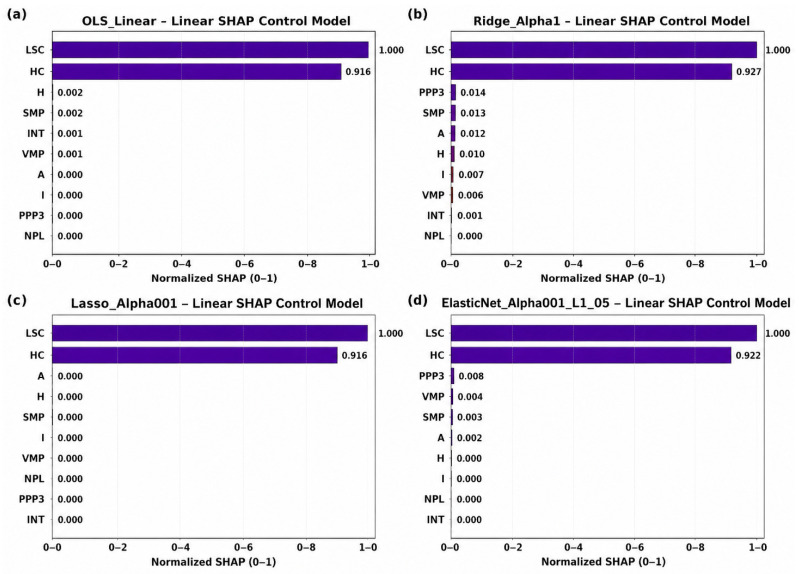
Comparison of normalized LinearSHAP feature importance across OLS, Ridge, Lasso, and Elastic Net baseline models. (**a**) OLS_Linear, (**b**) Ridge_Alpha1, (**c**) Lasso_Alpha001, (**d**) ElasticNet_Alpha001_L1_05.

As depicted in Figure 17, there is very high consistency in the ranking of feature importances among all four linear baseline models. The quality of the living standards (LSC) continues to be the main predictor in each specification and has normalized SHAP values of 1.000 under OLS, Ridge, Lasso, and Elastic Net. The assessment of the health conditions (HC) maintains its position in second place, having normalized SHAP values of 0.916 for OLS, 0.927 for Ridge, 0.916 for Lasso, and 0.922 for Elastic Net. The gap between these two variables and other predictors clearly shows that the key factors influencing the MPI are independent of the linear regression approach applied.

Apart from the two prominent predictors, the linear models demonstrate significant discrepancies regarding the contribution from secondary variables. Specifically, the OLS estimation indicates the extremely low normalized SHAP coefficients of all the remaining predictors, ranging from human capital (H)—0.002 to the remaining variables—0.001 or less, with internet access (INT) having an impact of 0.001. Ridge Regression, on the other hand, offers slightly higher contribution estimates from a few secondary socioeconomic variables, such as PPP3 (0.014), SMP (0.013), assets (A) (0.012), human capital (H) (0.010), income (I) (0.007), and VMP (0.006). In turn, Elastic Net estimates similarly modest contributions from PPP3 (0.008), VMP (0.004), SMP (0.003), and assets (0.002), eliminating the contribution from other predictors via coefficient shrinkage. Lasso, however, uses variable selection strategy and attributes zero SHAP importance to all predictors apart from LSC and HC, showing that those two dimensions explain almost all of the linearly predictable information in the dataset.

As can be seen from Figure 17, where comparative analysis is provided, despite the difference in the level of coefficient regularization in the four linear algorithms, they are united in recognizing living standards and health conditions as the key structural factors driving multidimensional poverty. However, more importantly, the linear model is not able to capture any significant explanatory role of other variables, which implies the inability of traditional linear methods to reflect adequately the complicated structure of interdependencies in the MPI system. In comparison with these initial models, the MLP-KernelSHAP approach offers an explanation with significantly more depth.

In general, this sensitivity analysis based on the linear baseline control confirms that the key insights generated by this paper are extremely robust to different model specifications. The fact that LSC and HC emerge again as the two most important predictors for OLS, Ridge, Lasso, and Elastic Net, as well as nonlinear MLP, suggests that the results obtained cannot be seen as an artifact of using any particular model but rather as stable structural properties of multidimensional poverty.

## 4. Discussion

According to the empirical findings, living standards (LSC) and health conditions (HC) are the most powerful structural factors determining educational deprivation. On the other hand, income factors reveal relatively weaker direct impacts. Such findings correspond well with multidimensional poverty analysis where education has been identified to be related more to basic service deprivations and poor health rather than income [2,8].

Poor health will lower children’s school participation rates and their capacity to learn. In addition, the lack of access to adequate infrastructure, such as electricity, proper sanitation facilities, decent accommodation, and other facilities, makes it difficult for learners to acquire knowledge effectively. The above mechanisms explain why structural deprivations have more impact on education than economic factors.

The SHAP interaction network has been identified as partially fragmented. Instead of showing independent poverty dimensions, fragmentation means that there are pockets of highly interconnected clusters with less connectivity at the overall level. This means that poverty is exacerbated by localized effects, meaning that any strategy directed toward the key structural aspects can positively affect the whole multidimensional structure of poverty.

As far as the methodology goes, it is worth noting that the exceptionally high explanatory power achieved by the MLP model (R^2^ = 0.9847) cannot be considered an instance of out-of-sample predictive accuracy according to classical econometric definition. Indeed, such an outcome stems directly from the very nature of the mathematical construction of the Alkire–Foster MPI framework, where indicators are mutually interrelated and generated using deterministic functions. Thus, one can say that MLP serves as a means of extracting inherent dependencies between MPI components and not as a poverty outcome predictor.

Within this setting, the modeling process becomes essentially concerned with unveiling the hidden dependency structures of MPI components, thus turning it into a process of analyzing the underlying construction of the index.

By incorporating machine learning algorithms, SHAP values, network theory and entropy, the approach presented expands the scope of application of explainable AI techniques by providing a unified description of the dependency structures of MPI variables.

However, the suggested approach may be subjected to several constraints depending on certain conditions. First, in case the dataset contains a relatively large number of explanatory variables per observation, the SHAP-based network may become unreliable due to the presence of multicollinearity and feature redundancies, thus resulting in the possible bias of feature importance calculations. Second, in case the underlying data structure deviates from the deterministic nature of the Alkire–Foster framework (e.g., microlevel datasets that are independent and/or contain a lot of noise), the explanatory ability of the selected nonlinear models (e.g., MLP) might become rather limited since the current framework relies heavily on the interdependencies present in the MPI components. Third, small country-level samples (N = 109) may reduce the generalization capabilities of the considered deep nonlinear models, making them susceptible to sample variations and parameter tuning. Finally, feature sparsity and an insufficient interaction effect in certain datasets may render both network- and spectrum-based explanations unstable. Overall, the current framework can be successfully employed in structured socioeconomic systems like MPI.

## 5. Conclusions

In this paper, a novel Explainable Artificial Intelligence approach was suggested that involved the use of neural networks, SHAP analysis, network science, spectral graph theory, and entropy analysis with the aim to analyze the structural complexity of multidimensional poverty. The results suggest that living conditions and state of health are the leading structural factors affecting educational poverty, and the suggested methodology can help visualize the nonlinear relations within the MPI framework.

In contrast to other studies focusing on the predictive aspects, the novelty of this research lies in its focus on discovering the structural and topological dependence within the multidimensional poverty framework. The combination of explainable AI and spectral–entropy analysis within networks is the primary methodological innovation.

Several limitations are associated with this paper. Firstly, the approach uses cross-sectional country-level data; thus, the obtained findings are not to be considered predictive but rather structural. Additionally, the use of structurally-dependent Alkire–Foster index measures make results non-predictive. Secondly, due to the limited sample size, it becomes hard to train complex neural networks.

Future studies need to use household-level microdata (such as DHS or MICS), longitudinal data, and other explainable machine learning methods.

## 6. Policy Implications

The findings imply that any policy efforts that focus solely on increasing incomes will not have much success in reducing educational deprivations. Policy makers need to devise integrated strategies targeting the various structural aspects highlighted from the findings of the SHAP interaction analysis.

One such policy recommendation will entail a mix of provision of healthcare in schools, health nutrition, improved sanitation, reliable electricity supply, and improvements in housing standards in poor neighborhoods. Since the health and living standards category has been revealed to have the highest interactions among all categories, joint efforts will lead to more gains in educational performance.

Generally, the findings suggest that education policies should be developed in combination with health and living standards and not as standalone initiatives.

## Figures and Tables

**Table 1:** Operational definitions of the variables used in the study.

Variable	Definition	Indicator/Unit	Role
MPI	Multidimensional Poverty Index	Index (0–1)	Overall poverty measure
H	Headcount Ratio	Percentage of multidimensionally poor population (%)	Poverty incidence
A	Intensity of Deprivation	Average deprivation among poor (%)	Poverty intensity
I	Inequality among the Poor	MPI inequality measure	Explanatory variable
SMP	Severe Multidimensional Poverty	Percentage (%)	Explanatory variable
VMP	Vulnerable to Multidimensional Poverty	Percentage (%)	Explanatory variable
HC	Health Component	Weighted deprivation score	Explanatory variable
EC	Education Component	Weighted deprivation score	Dependent variable
LSC	Living Standards Component	Weighted deprivation score	Explanatory variable
NPL	Population below National Poverty Line	Percentage (%)	Explanatory variable
PPP3	Population living below PPP $3/day	Percentage (%)	Explanatory variable

**Table 2:** Model training parameters and reproducibility settings.

Model	Hyperparameter Search Space/Fixed Settings	Optimization Strategy
SVR	C ∈ [1,20], ε ∈ [0.01,0.5], kernel = RBF (default), γ = scale	RandomizedSearchCV (n_iter = 10, CV = 5)
k-NN	n_neighbors ∈ [3–14], weights ∈ {uniform, distance}	RandomizedSearchCV (n_iter = 10, CV = 5)
MLP	hidden_layer_sizes ∈ {(100,50), (120,60), (150,80)}, α ∈ {0.0001, 0.001}, max_iter = 2000, activation = ReLU (default), solver = Adam (default)	RandomizedSearchCV (n_iter = 10, CV = 5)
Decision Tree	max_depth ∈ {3,5,10,None}, random_state = 42	RandomizedSearchCV (n_iter = 10, CV = 5)
Random Forest	n_estimators ∈ {200,300}, max_depth ∈ {None,10}, random_state = 42	RandomizedSearchCV (n_iter = 10, CV = 5)
Extra Trees	n_estimators ∈ {200,300}, random_state = 42	RandomizedSearchCV (n_iter = 10, CV = 5)
Gradient Boosting	n_estimators ∈ {200,300}, learning_rate ∈ {0.05,0.1}, random_state = 42	RandomizedSearchCV (n_iter = 10, CV = 5)
AdaBoost	n_estimators ∈ {200,300}, random_state = 42	RandomizedSearchCV (n_iter = 10, CV = 5)
Bagging	n_estimators ∈ {50,100}, random_state = 42	RandomizedSearchCV (n_iter = 10, CV = 5)
XGBoost	n_estimators ∈ {200,300}, max_depth ∈ {3,5}, verbosity = 0, random_state = 42	RandomizedSearchCV (n_iter = 10, CV = 5)
LightGBM	n_estimators ∈ {200,300}, num_leaves ∈ {30,50}, random_state = 42	RandomizedSearchCV (n_iter = 10, CV = 5)
CatBoost	iterations ∈ {200,300}, depth ∈ {4,6}, verbose = 0, random_state = 42	RandomizedSearchCV (n_iter = 10, CV = 5)
Stacking	Base models: RF(200), XGB(200), LGBM(200); Meta-model: Ridge Regression	Fixed ensemble (no tuning)

## Data Availability

The dataset and Python codes used in this paper can be accessed at the following address: https://github.com/Sadullah4535/Multidimensional-Poverty-Indicators- (accessed on 25 March 2026).
